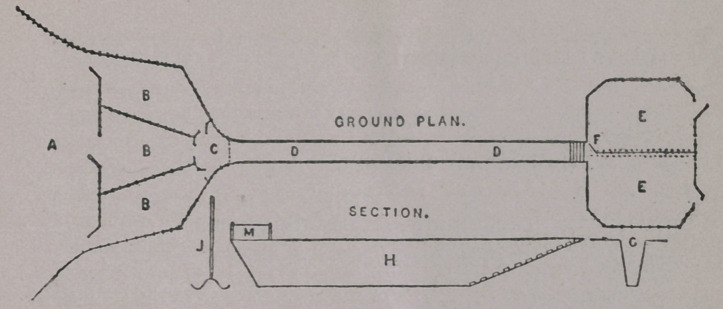# A Dipping Vat for Sheep

**Published:** 1891-09

**Authors:** 


### A DIPPING VAT FOR SHEEP.*

We are indebted to Messrs. Cooper, the well-known sheep-dip manufacturers, for the following description of a swimming bath greatly used in Australia for dipping large flocks of sheep:—

### HOW TO BUILD A DIPPING VAT.

These suggestions, the result of practical experience in all parts of the world, are only intended to convey some idea of the general method of arranging a suitable dipping station. The details can of course be varied to suit individual requirements.

* From the *Agricultural Journal*, Cape Colony, Africa.

A. Mustering inclosure into which the sheep to be dipped are collected.

BBB. Pens by means of which the sheep are conveyed a few at a time from the mustering inclosure to the small internal pen C, in which a man is placed to pass them singly into the bath.

DD. The bath or swim, usually fifty feet long, five feet deep, twenty-one inches wide at the top, tapering to six inches wide at bottom, as shown in section G. For a short distance along the sides at the entrance end of the bath a board M, two feet high, should be fixed to catch the splash. Towards the end at which the sheep leave the bath the bottom should rise gradually, with ribbed foothold, to the level of the draining pens as shown in section H. This will greatly assist the sheep in getting out, besides economizing wash.

To avoid having to constantly measure the water when replenishing the wash, it is well to have a guage board fixed in the bath and marked plainly at frequent intervals to indicate from time to time the number of gallons which are added.

EE. The drawing pens. These are filled alternately by means of a swing gate F which serves for both pens. Each pen should be large enough to hold not less than 200 or 250 sheep.

A great saving of wash will be effected if the floors of these pens be made slightly sloping from the outside towards the dividing fence, into a gutter, to conduct the drippings back to the bath.

J. Crutch for pressing the backs of the sheep under the wash as they swim along the bath. It can also be used to assist any that are too weak to swim.

Never build a shorter bath than is above suggested whatever Dip may be used.

## Figures and Tables

**Figure f1:**